# The distal femur is a reliable guide for tibial plateau fracture reduction: a study of measurements on 3D CT scans in 84 healthy knees

**DOI:** 10.1186/s13018-018-0933-8

**Published:** 2018-09-04

**Authors:** Sorawut Thamyongkit, Laura M. Fayad, Lynne C. Jones, Erik A. Hasenboehler, Norachart Sirisreetreerux, Babar Shafiq

**Affiliations:** 1 Department of Orthopaedic Surgery, The Johns Hopkins University, 4940 Eastern Avenue, Baltimore, MD 21224 USA; 2 Chakri Naruebodindra Medical Institute, Faculty of Medicine Ramathibodi Hospital, Mahidol University, 270 Rama VI Road, Ratchatewi, Bangkok, 10400 Thailand; 3 Russell H. Morgan Department of Radiology and Radiological Science, The Johns Hopkins University, 601 North Caroline Street, Baltimore, MD 21224 USA; 4 Department of Orthopaedic Surgery, The Johns Hopkins University, 601 N. Caroline St., Fl. 5, Baltimore, MD 21205 USA

**Keywords:** Femoral condyle articular width, Knee, Reduction, Tibial articular width, Tibial plateau fracture

## Abstract

**Background:**

Limited data have been published regarding the typical coronal dimensions of the femur and tibia and how they relate to each other. This can be used to aid in judging optimal operative reduction of tibial plateau fractures. The purpose of the present study was to quantify the width of tibial plateau in relation to the distal femur.

**Methods:**

We reviewed 3D computed tomography (CT) scans taken between 2013 and 2016 of 42 patients (84 knees). We measured positions of the lateral tibial condyle with respect to the lateral femoral condyle (dLC) and the medial tibial condyle with respect to the medial femoral condyle (dMC) in the coronal plane. Positions of the articular edges of the lateral and medial tibia were also measured with respect to the femur (dLA and dMA).

**Results:**

The mean (± standard deviation) measurements were as follows: dLC, − 0.1 ± 1.9 mm; dMC, − 4.7 ± 4.1 mm; dLA, 0.9 ± 1.0 mm; and dMA, 0.1 ± 1.5 mm. The mean (± standard deviation) ratio of tibial to femoral condylar width was 0.91 ± 0.03, and the ratio of tibial to femoral articular width was 1.01 ± 0.04.

**Conclusions:**

The articular width of the tibia laterally and medially was slightly wider than the femoral articular width. These small differences and deviations indicate that the femur might be used as a reference to judge tibial plateau width reduction.

## Background

Surgical outcomes after tibial plateau fracture are associated strongly with functional alignment, knee range of motion, and stability [1, 2]. The primary goals of tibial plateau fracture treatment are to restore the articular surface, sagittal and coronal alignment, and condylar width. Splitting of the lateral plateau causes a loss of lateral support for the lateral femoral condyle, leading to coronal instability and osteoarthritis [3]. Honkonen [4] found that condylar width is a predictor of clinical outcome. Residual increase in tibial condylar width after treatment tends to affect outcomes adversely [4, 5]. Currently, the most common reference for intraoperative tibial condylar width reduction is the ipsilateral femur. Surgeons typically will drop a plumb line from the lateral femoral condyle and aim for reduction of the tibial plateau to within this line. Another goal is to align the lateral tibial articular edge with the femoral articular edge. Limited data have been published regarding the typical coronal dimensions of the femur and tibia and how they relate to each other with respect to condylar and articular width [6–8].

The purpose of this study was to quantify the relationship between measures of the distal femur and those of the proximal tibia such that they can be used to aid in judging optimal operative reduction of tibial plateau fractures. The reliability of three different means of determining anterior-posterior (AP) view was also assessed. Our hypotheses were that (1) radiographic measurements of the distal femur would correspond reliably to those of the proximal tibia and could be used during surgery to judge fracture reduction and (2) that the posterior condylar tangent line view would provide the most reproducible AP view.

## Methods

### Patients

**Fig. 1:**
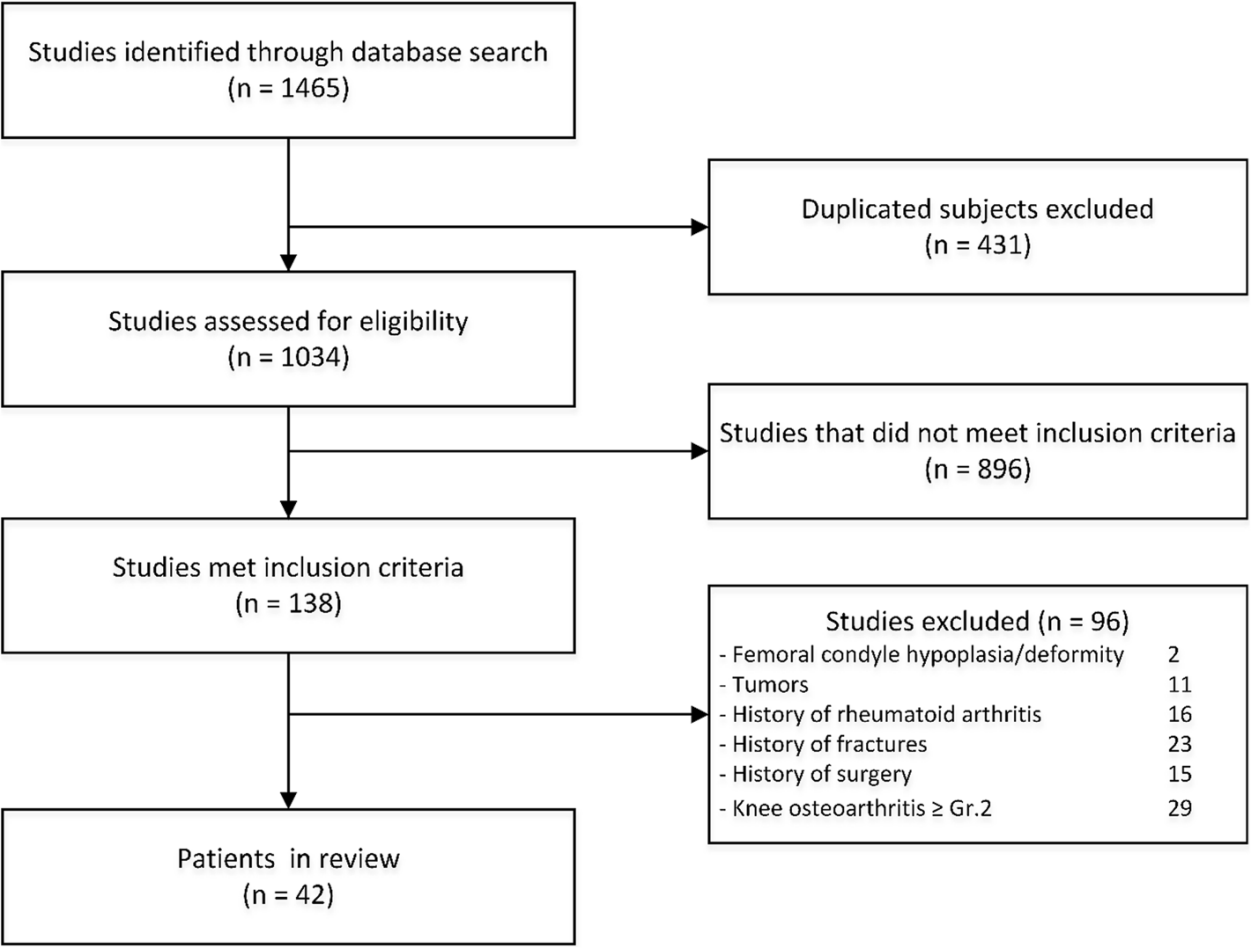
Flow diagram showing selection of 42 patients who underwent knee CT scans for nontraumatic reasons between June 2013 and May 2016

This is a retrospective review study (level of evidence: III). After obtaining institutional review board approval (IRB00105744), we reviewed computed tomography (CT) studies of patients who underwent knee CT. A total of 1465 CT studies, taken between June 2013 and May 2016, were available. We included patients aged 18–65 years who had CT scans of both knees for nontraumatic reasons, resulting in 138 patients. We excluded 96 patients who had femoral condyle hypoplasia or periarticular abnormalities such as tumors, rheumatoid arthritis, a history of fracture around the knee, a history of proximal tibia or distal femur surgery, or radiographic evidence of knee osteoarthritis of grade 2 or higher (according to the Kellgren and Lawrence classification [9]). CT images of 84 healthy knees in 42 patients (23 women) with a mean (± standard deviation) age of 48 ± 15 years were analyzed (Fig. 1).

### CT technique

CT scans were performed using the Flash or Somatom Sensation 64 (Siemens Medical Systems, Malvern, PA). Images were exported to a picture archiving and communication system with three-dimensional (3D) postprocessing software (Carestream Vue PACS, Carestream Health, Rochester, NY). 3D models of the knee were created from thin-section, 0.75-mm axial cuts. All measurements were made on the resultant models.

3D CT imaging was used instead of plain radiographs, because all images could be rotated reproducibly to anterior-posterior (AP) views commonly used intraoperatively. Image rendering and image transparency were then adjusted such that two-dimensional images similar to those seen fluoroscopically were used for measurements.

### Measurements

**Fig. 2:**
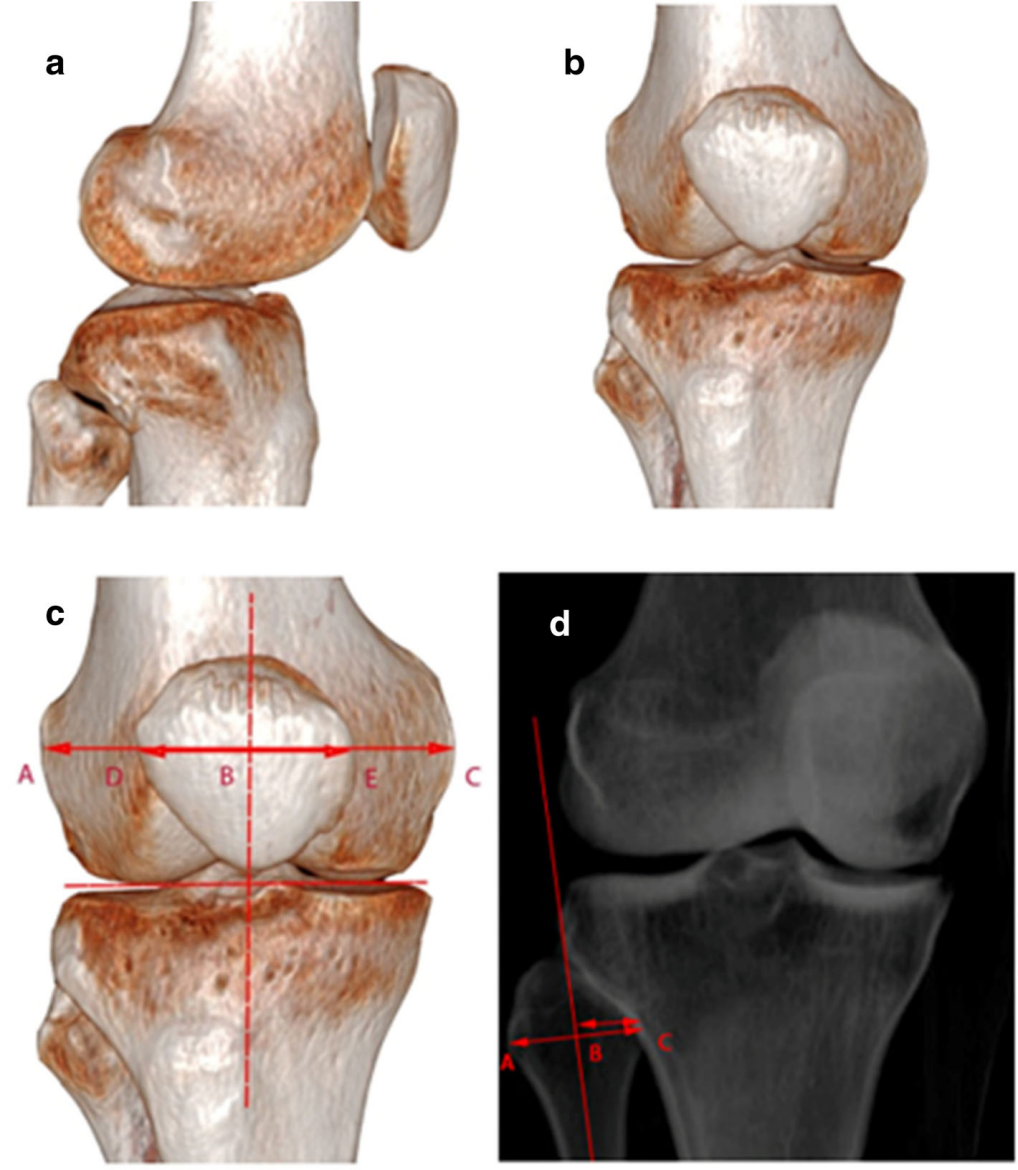
**a** Lateral view defined as a view that has posterior femoral condyles superimposed precisely. **b** Posterior condylar tangent line anterior-posterior view defined as perpendicular (90°) to the lateral view of the distal femur. **c** Patella-in-center anterior-posterior view defined as a coronal view of the knee with the patella centered between the distal femoral condyles. **d** Fibular coverage anterior-posterior view defined as a coronal view of the knee with 50% overlap of the fibular head with the tibia

All measurements were taken in the coronal view to simulate an AP view by fluoroscopy. The following reference methods were used to define coronal views: (1) patella-in-center (PIC-AP) view, which was a coronal view of the knee with the patella centered between the distal femoral condyles; (2) fibular coverage (FC-AP) view, which was a coronal view of the knee with 50% overlap of the fibular head; and (3) posterior condylar tangent line (PCT-AP) view, which was 90° to the true lateral view of the distal femur (true lateral view of the distal femur was defined as the view that shows complete superimposition of the posterior aspects of the femoral condyles) (Fig. 2).

**Fig. 3:**
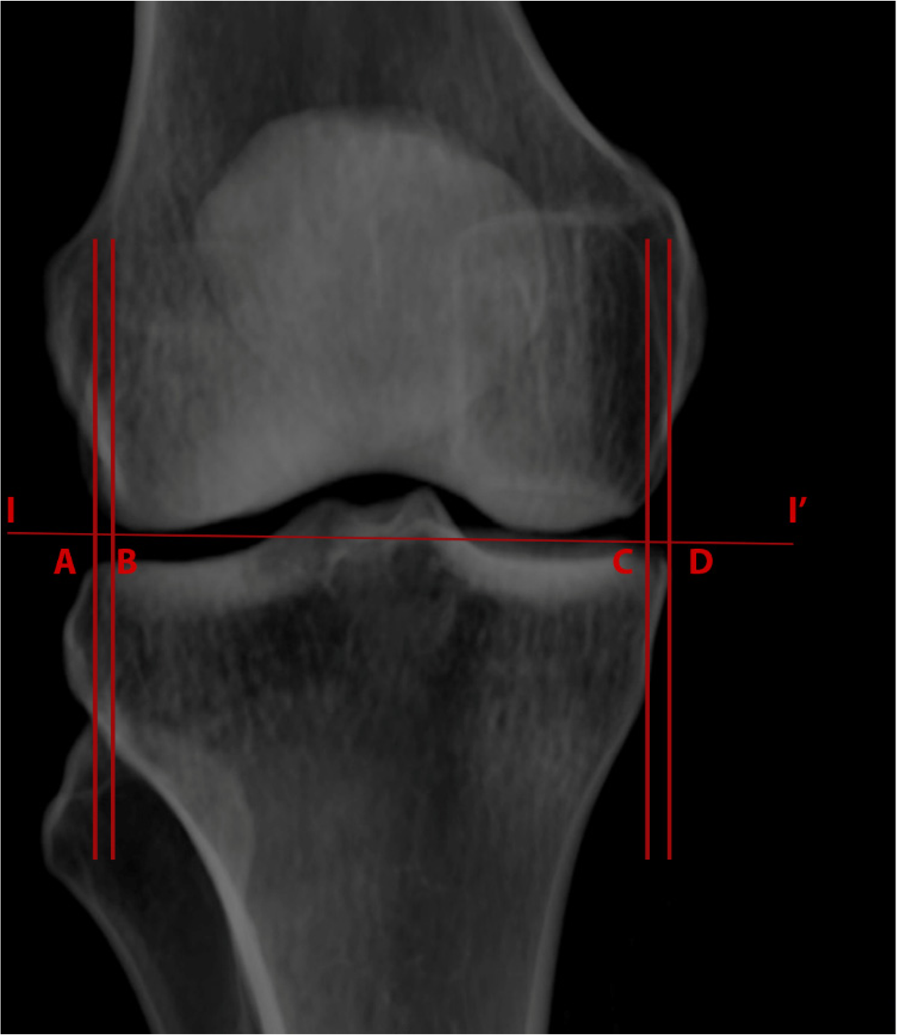
Knee measurements. A–B, horizontal distance between lateral femoral articular edge and lateral tibial articular edge (dLA); C–D, horizontal distance between medial femoral articular edge to medial tibial articular edge (dMA); I–I′, distal femoral joint line

**Fig. 4:**
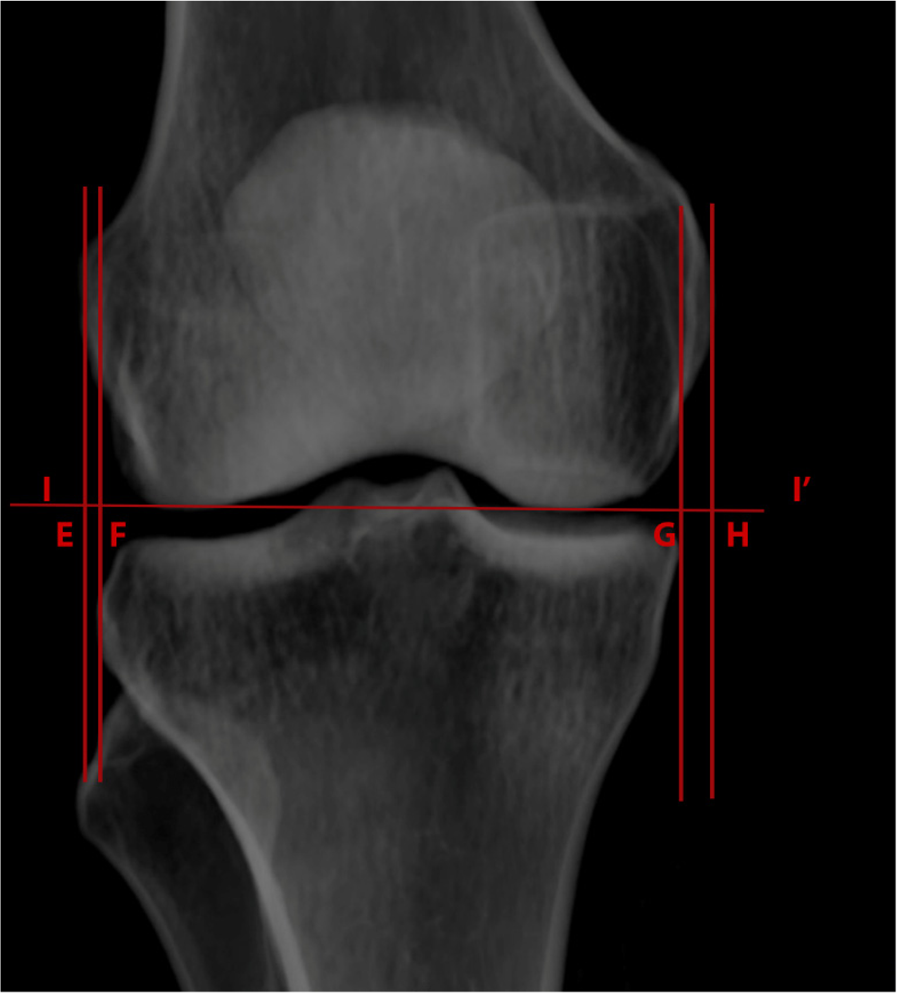
Knee measurements. E–F, horizontal distance between lateral femoral condyle edge and lateral tibial condyle edge (dLC); G–H, horizontal distance between medial femoral condyle edge to medial tibial condyle edge (dMC); I–I′, distal femoral joint line

The following six comparisons were made and recorded in millimeters (Figs. 3 and 4): dLA, horizontal distance from the lateral femoral articular edge to the lateral tibial articular edge; dLC, horizontal distance from the lateral femoral condyle to the lateral tibial condyle; dMA, horizontal distance from the medial femoral articular edge to the medial tibial articular edge; dMC, horizontal distance from the medial femoral condyle to the medial tibial condyle; percent of fibular coverage by tibia on PIC-AP and PCT-AP; and tibial to femoral articular width ratio.

The first 20 patients’ images were evaluated independently twice, at 2-week intervals, by the senior author, the first author, and one additional author to determine inter- and intraobserver reliability. The measurement methods are detailed in Table 1.

**Table 1:** Description of distal femur and proximal tibia measurements

Measure	Description
Distance	
Lateral femoral articular edge to lateral tibial articular edge (dLA)	Horizontal distance between the most lateral femoral articular edge to the most lateral tibial articular edge, parallel to the joint line, on coronal view.^1^
Lateral femoral condyle to lateral tibial condyle (dLC)	Horizontal distance between the most lateral femoral condyle to the lateral tibial condyle, parallel to the joint line, on coronal view.^2^
Medial femoral articular edge to medial tibial articular edge (dMA)	Horizontal distance between the most medial femoral articular edge to the most medial tibial articular edge, parallel to the joint line, on coronal view.^3^
Medial femoral condyle to medial tibial condyle (dMC)	Horizontal distance between the most medial femoral condyle to the medial tibial condyle, parallel to the joint line, on coronal view.^4^
Articular width ratio	Tibial articular width (distance between the most medial to the most lateral of tibial articular edge, parallel to the joint line, on coronal view) divided by femoral articular width (distance between the most medial to the most lateral of femoral articular edge, parallel to the joint line, on coronal view)
Percentage	
Fibular coverage in PIC-AP view	Percentage of fibular coverage by tibia on coronal view. The widest aspect of the fibular head was measured perpendicular to the fibular axis. A vertical line was made at the most lateral edge of the tibia and parallel to the fibular axis. Fibular coverage was defined as percentage of fibular head width transected to the vertical line from the tibial edge. Coronal view was defined as patella-in-center between the medial and lateral epicondyle of distal femur.
Fibular coverage in PCT-AP view	Percentage of fibular coverage by tibia on coronal view. Widest aspect of fibular head was measured perpendicular to the fibular axis. A vertical line was made at the most lateral edge of the tibia and parallel to the fibular axis. Fibular coverage was defined as percentage of fibular head width transected to the vertical line from the tibial edge. Coronal view was defined as 90° to the lateral view of the distal femur (posterior aspects of the femoral condyles are superimposed).

*AP* anterior-posterior, *PCT* posterior condylar tangent, *PIC* patella-in-center

^1^A positive value means the lateral tibial articular edge is more lateral compared with the femur; a negative value means the femoral articular edge is more lateral compared with the tibia

^2^A positive value means the lateral tibial condyle is more lateral compared with the femur; a negative value means the lateral femoral condyle is more lateral compared with the tibia

^3^A positive value means the medial tibial articular edge is more medial compared with the femur; a negative value means the medial femoral articular edge is more medial compared with the tibia

^4^A positive value means the medial tibial condyle is more medial compared with the femur; a negative value means the femoral condyle is more medial compared with the tibia

### Statistical analysis

According to the results of a study by Mensch and Amstutz [10], the required sample size was determined to be 35 subjects. This sample size allowed detection of a difference of 3 mm with 80% power and a two-sided *α* value of 0.05. Inter- and intraobserver correlation was evaluated to determine reliability using intraclass correlation coefficients (ICCs) (values greater than 0.8 are considered excellent agreement). Categorical data were analyzed using the Fisher exact test. Continuous data were summarized as means and standard deviations and analyzed using Student *t* tests or analysis of variance.

## Results

The inter- and intraobserver reliability of measurements on the three coronal views were evaluated. The PCT-AP view had more good-to-excellent interobserver ICC values (> 0.6) than the PIC-AP and FC-AP views (*p* = 0.012) (Table 2). Although intraobserver ICC values were not significantly different between views, most intraobserver ICC values were good-to-excellent in PCT-AP views. Therefore, we chose to analyze measurements performed using the PCT-AP view.

**Table 2:** Intraclass correlation coefficients for interobserver and intraobserver reliability in three AP views

Measure	Interobserver reliability			*p*	Intraobserver reliability			*p*
	PCT-AP	PIC-AP	FC-AP		PCT-AP	PIC-AP	FC-AP	
dMC	0.71	0.61	0.50	0.012	0.67	0.57	0.63	0.253
dLC	0.84	0.44	0.44		0.70	0.81	0.76	
dMA	0.75	0.59	0.46		0.72	0.30	0.60	
dLA	0.75	0.51	0.20		0.47	0.13	0.25	

Values greater than 0.6 are considered good-to-excellent agreement

*AP* anterior-posterior, *dLA* distance from lateral femoral articular edge to lateral tibial articular edge, *dLC* distance from lateral femoral condyle to lateral tibial condyle, *dMA* distance from medial femoral articular edge to medial tibial articular edge, *dMC* distance from medial femoral condyle to medial tibial condyle, *FC* fibular coverage

Using the PCT-AP view, the mean ratio of tibial to femoral condylar width was 0.91 ± 0.03, and the ratio of tibial to femoral articular width was 1.01 ± 0.04. This demonstrates that the overall femoral condyle width is slightly greater than the tibia, whereas the overall tibial articular width is slightly greater than the femur.

The mean dLC was − 0.1 ± 1.9 mm, and the mean dLA was 0.9 ± 1.0 mm. This demonstrates that when looking at the lateral side only, the lateral tibial condyle is slightly smaller than the femur, whereas the lateral tibial articular edges are wider than the femur.

The percentages of the fibula covered by the tibia in both PIC-AP and PCT-AP views were more than 50% (56% ± 11% and 67% ± 12%, respectively) (Table 3). This suggests that the 50% fibula coverage view does not correspond reliably to the most reproducible AP view, namely the PCT-AP view, and should therefore not be used as a method of judging a good AP view.

**Table 3:** Morphologic measurements of 84 knees in 42 patients

Measure	Total	Side		*p* value	Sex		*p* value
		Left	Right		Male	Female	
Distance, mm							
dMC	− 4.7^a^ (4.1)	− 4.5 (3.0)	− 5.0 (5.2)	0.583	− 0.6 (0.6)	− 0.4 (0.2)	0.082
dLC	− 0.1^b^ (1.9)	− 0.9 (1.7)	− 0.9 (2.1)	0.958	0.0 (0.2)	− 0.1 (0.2)	0.317
dMA	0.1 (1.5)	0.0 (1.3)	0.2 (1.4)	0.443	0.0 (0.2)	0.0 (0.1)	0.076
dLA	0.9 (1.0)	1.0 (1.0)	0.8 (1.1)	0.408	0.1 (0.1)	0.1 (9.8)	0.836
Fibular coverage, %							
PIC-AP	56 (11)	56 (10)	57 (12)	0.860	59 (12)	54 (10)	0.295
PCT-AP	67 (12)	67 (12)	67 (12)	0.887	69 (13)	65 (2.4)	0.073
Articular width ratio	1.01 (0.04)	1.01 (0.04)	1.02 (0.05)	0.231	1.01 (0.05)	1.02 (0.04)	0.079

The negative values for the tibia indicate that the tibia was narrower than the femur in the coronal plane. Data are the mean, and data in parenthesis are standard deviation

*AP* anterior-posterior, *dLA* distance from lateral femoral articular edge to lateral tibial articular edge, *dLC* distance from lateral femoral condyle to lateral tibial condyle, *dMA* distance from medial femoral articular edge to medial tibial articular edge, *dMC* distance from medial femoral condyle to medial tibial condyle

^a^The medial femoral condyle was medial to the medial tibial condyle

^b^The lateral femoral condyle was lateral to the lateral tibial condyle

## Discussion

The most reproducible AP view for making anatomic measurements was the PCT-AP view, which had higher inter- and intraobserver reliability than measurements taken in the PIC-AP and FC-AP views. Thus, we decided to analyze and report all measurements on the PCT-AP view. The results show that the contralateral tibial plateau and the ipsilateral distal femur might conceivably be used as references for tibial plateau fracture reduction. We found that the lateral articular edge of the tibia was slightly lateral to the lateral articular edge of the femur (mean dLA, 0.9 ± 1 mm). This suggests that attempting to reduce the lateral tibial condylar or articular edges to within the margins of the corresponding femoral edges will result in over-reduction. Thus, in fracture reduction, the lateral tibial plateau articular edge should be slightly more lateral and not medial to the lateral articular edge of the distal femur. Similarly, the medial articular edge of the tibia should be slightly more medial and not lateral to the medial articular edge of the femur (mean dMA, 0.1 ± 1.5 mm). Although the size of the distal femur and proximal tibia may differ between men and women, there were no significant differences in dMC, dLC, dMA, and articular width ratio between men and women. Understanding these anatomic relationships may help orthopedic surgeons improve the accuracy of tibial plateau fracture reduction during surgery.

Previous studies found that an increase in the original width of the tibial plateau by more than 5 mm, or 105% compared with the femoral condylar width, is related to long-term development of degenerative lesions of the meniscus and poor outcomes [4, 5]. During tibial plateau fracture surgery, many surgeons use the ipsilateral femoral condyle or contralateral tibial plateau width as the reference width [11–17]. The contralateral tibial plateau may be more suitable as a template for repair of tibial plateau fractures because surgeons typically assume the two sides are identical. However, unequal size of the distal femoral width for each specimen in a cadaveric study has been reported [18] and may indicate a possibility of unequal size of the tibial plateaus in each individual. The drawbacks of using the contralateral knee radiograph to assess reduction are the additional radiation exposure to patients and the need for additional measurements for comparison. Conversely, using the distal femur as a reference does not require a second radiograph and could be a simple landmark for reduction of the proximal tibial plateau fracture.

One explanation for the discrepancy in reliability is that the PIC-AP view depends on subjective assessment of the position of the patella in the trochlea. In the FC-AP view, the percentage of fibular coverage was higher when the knee had more external rotation and lower when the knee had more internal rotation. Thus, the FC-AP view had different rotation depending on each subject’s anatomy and may not have been 90° to the lateral view. In our study, PIC-AP and FC-AP views had more internal rotation compared with PCT-AP. These results support the recommendation that surgeons use the PCT-AP view because measurements taken in this view are more reliable and reproducible than those taken in the PIC-AP and FC-AP views.

There are several limitations to this study. First, we included a small number of patients (84 knees in 42 subjects) because we included only patients who had bilateral CT scans of healthy knees. Significant differences may be found in a larger study population. However, the magnitude of the difference in these measurements is likely to be small. Second, these measurements may be difficult to apply in patients who have deformity or osteoarthritic changes of the knee. Third, we used 3D CT imaging to represent AP views of two-dimensional images. A prospective study of measurements using intraoperative fluoroscopic images is needed to validate these results.

## Conclusions

For intraoperative tibial plateau width reduction, the ipsilateral femoral condyle articular width is one of the reliable references. Importantly, both articular edges of the femur and tibia were almost overlapping or coincident on the AP view. The mean lateral tibial articular edge is slightly more lateral than the lateral femoral articular edge. These results support the recommendation that surgeons use the ipsilateral distal femur as one reference to guide proximal tibial fracture reduction.

## Abbreviations

2D: Two-dimensional
3D: Three-dimensional
AP: Anterior-posterior
CT: Computed tomography
dLA: Distance from lateral femoral articular edge to lateral tibial articular edge
dLC: Distance from lateral femoral condyle to lateral tibial condyle
dMA: Distance from medial femoral articular edge to medial tibial articular edge
dMC: Distance from medial femoral condyle to medial tibial condyle
FC: Fibular coverage
PCT: Posterior condylar tangent
PIC: Patella-in-center

## References

[1] Barei DP, Nork SE, Mills WJ (2006). Functional outcomes of severe bicondylar tibial plateau fractures treated with dual incisions and medial and lateral plates. J Bone Joint Surg Am.

[2] Malakasi A, Lallos SN, Chronopoulos E (2013). Comparative study of internal and hybrid external fixation in tibial condylar fractures. Eur J Orthop Surg Traumatol.

[3] Rasmussen PS (1973). Tibial condylar fractures. Impairment of knee joint stability as an indication for surgical treatment. J Bone Joint Surg Am.

[4] Honkonen SE. Indications for surgical treatment of tibial condyle fractures. Clin Orthop Relat Res. 1994;302:199–205. 8168301

[5] Mattiassich G, Foltin E, Pietsch M (2015). Magnetic resonance evaluation in long term follow up of operated lateral tibial plateau fractures. BMC Musculoskelet Disord.

[6] OF E, Kucukdurmaz F, Sayar S (2016). Anthropometric measurements of tibial plateau and correlation with the current tibial implants. Knee Surg Sports Traumatol Arthrosc.

[7] Shah DS, Ghyar R, Ravi B, Shetty V (2013). 3D morphological study of the Indian arthritic knee: comparison with other ethnic groups and conformity of current TKA implant. OJRA.

[8] Yue B, Varadarajan KM, Ai S (2011). Differences of knee anthropometry between Chinese and white men and women. J Arthroplast.

[9] Kellgren JH, Lawrence JS (1957). Radiological assessment of osteo-arthrosis. Ann Rheum Dis.

[10] Mensch JS, Amstutz HC. Knee morphology as a guide to knee replacement. Clin Orthop Relat Res. 1975;112:231–41. 1192638

[11] Ballmer FT, Hertel R, Notzli HP (2000). Treatment of tibial plateau fractures with small fragment internal fixation: a preliminary report. J Orthop Trauma.

[12] Dall'oca C, Maluta T, Lavini F (2012). Tibial plateau fractures: compared outcomes between ARIF and ORIF. Strategies Trauma Limb Reconstr.

[13] El Barbary H, Abdel Ghani H, Misbah H, Salem K (2005). Complex tibial plateau fractures treated with Ilizarov external fixator with or without minimal internal fixation. Int Orthop.

[14] Hsu CJ, Chang WN, Wong CY (2001). Surgical treatment of tibial plateau fracture in elderly patients. Arch Orthop Trauma Surg.

[15] Kayali C, Ozturk H, Altay T (2008). Arthroscopically assisted percutaneous osteosynthesis of lateral tibial plateau fractures. Can J Surg.

[16] Ramos T, Ekholm C, Eriksson BI (2013). The Ilizarov external fixator--a useful alternative for the treatment of proximal tibial fractures. A prospective observational study of 30 consecutive patients. BMC Musculoskelet Disord.

[17] Zhai Q, Hu C, Luo C (2014). Multi-plate reconstruction for severe bicondylar tibial plateau fractures of young adults. Int Orthop.

[18] Yoshioka Y, Siu D, TDV C (1987). The anatomy and functional axes of the femur. J Bone Joint Surg Am.

